# SIRT1-mediated downregulation of p27^Kip1^ is essential for overcoming contact inhibition of Kaposi's sarcoma-associated herpesvirus transformed cells

**DOI:** 10.18632/oncotarget.12359

**Published:** 2016-09-30

**Authors:** Meilan He, Hongfeng Yuan, Brandon Tan, Rosemary Bai, Heon Seok Kim, Sangsu Bae, Lu Che, Jin-Soo Kim, Shou-Jiang Gao

**Affiliations:** ^1^ Department of Molecular Microbiology and Immunology, Keck School of Medicine, University of Southern California Los Angeles, Los Angeles, CA, USA; ^2^ Center for Genome Engineering, Institute for Basic Science, Seoul, South Korea; ^3^ Department of Chemistry, Seoul National University, Seoul, South Korea; ^4^ Present address: Department of Chemistry, Hanyang University, Seoul, South Korea

**Keywords:** SIRT1, p27^Kip1^, Kaposi's sarcoma-associated herpesvirus, cellular transformation

## Abstract

Kaposi's sarcoma-associated herpesvirus (KSHV) is an oncogenic virus associated with Kaposi's sarcoma (KS), a malignancy commonly found in AIDS patients. Despite intensive studies in the last two decades, the mechanism of KSHV-induced cellular transformation and tumorigenesis remains unclear. In this study, we found that the expression of SIRT1, a metabolic sensor, was upregulated in a variety of KSHV-infected cells. In a model of KSHV-induced cellular transformation, SIRT1 knockdown with shRNAs or knockout by CRISPR/Cas9 gene editing dramatically suppressed cell proliferation and colony formation in soft agar of KSHV-transformed cells by inducing cell cycle arrest and contact inhibition. SIRT1 knockdown or knockout induced the expression of cyclin-dependent kinase inhibitor 1B (p27^Kip1^). Consequently, p27 knockdown rescued the inhibitory effect of SIRT1 knockdown or knockout on cell proliferation and colony formation. Furthermore, treatment of KSHV-transformed cells with a SIRT1 inhibitor, nicotinamide (NAM), had the same effect as SIRT1 knockdown and knockout. NAM significantly inhibited cell proliferation in culture and colony formation in soft agar, and induced cell cycle arrest. Significantly, NAM inhibited the progression of tumors and extended the survival of mice in a KSHV-induced tumor model. Collectively, these results demonstrate that SIRT1 suppression of p27 is required for KSHV-induced tumorigenesis and identify a potential therapeutic target for KS.

## INTRODUCTION

Infection by Kaposi's sarcoma-associated herpesvirus (KSHV) is required for the development of Kaposi sarcoma (KS) and several lymphoproliferative malignancies including primary effusion lymphoma (PEL) and a subset of multicentric Castleman's disease [1]. KS is a multifocal mesenchymal neoplasm characterized by hyperproliferative spindle-shaped tumor cells with frequent involvement of visceral organs. In Western countries, KS mostly occurs in the context of immunosuppression, especially in HIV-positive patients. The incidence of KS has stabilized following a decade of decline owing to the availability of anti-retroviral therapy. However, KS remains as one of the most important malignancies causing significant morbidity and mortality in some African regions [2]. There is currently no effective therapeutic treatment for KS, stressing the need for better understanding the development of KS and discovering novel therapeutic targets [3].

Most KS tumor cells are latently infected by KSHV, implicating that these cells are the driving force of KS. While numerous KSHV latent genes and microRNAs (miRNAs) have been shown to regulate cell proliferation and survival [4], the mechanism of KSHV-induced malignant proliferation remains unclear, which is in part due to the lack of a relevant model of KSHV-induced cellular transformation and tumorigenesis. We have recently shown that KSHV can efficiently infect and transform primary rat embryonic metanephric mesenchymal precursor (MM) cells [5]. KSHV-transformed MM cells (KMM) efficiently induce tumors in nude mice with virological and pathological features reminiscent of KS [5]. KMM cells are latently-infected by KSHV suggesting an essential role of viral latent genes in KSHV induced cellular transformation. This robust cell model has opened a new avenue for investigating the mechanism of KSHV-induced tumorigenesis. Using this model, we have shown that KSHV-encoded miRNAs and latent gene vFLIP (ORF71) are essential for KSHV-induced cellular transformation and tumorigenesis by regulating cell proliferation, survival and metabolic pathways [6, 7] while vCyclin (ORF72) promotes cellular transformation and tumorigenesis by overriding cell contact inhibition [8]. In addition, KSHV LANA (ORF73) promotes tumorigenesis by interacting with BMP-activated p-Smad1 to upregulate the expression of Id1 [9]. Despite these works, the cellular genes and pathways required for KSHV-induced transformation remain to be discovered.

We have previously shown that SIRT1 promotes KSHV latency by inhibiting the expression of the key activator for viral lytic replication RTA (ORF50) [10, 11]. In PEL cells, knockdown or inhibition of SIRT1 induced KSHV lytic replication. However, less than 10% of cells underwent lytic replication while most cells experienced crisis and eventually died, which indicated that SIRT1 might contribute to cell proliferation and survival in PEL cells.

SIRT1 is a multifaceted, highly conserved NAD^+^-dependent class III deacetylase involved in a wide variety of cellular processes including cancer [12, 13]. SIRT1 is a metabolic sensor and protects cells against metabolic, oxidative, and genotoxic stresses through deacetylation of multiple substrates including p53 [14], Ku70 [15], and FOXO [16, 17] proteins. The expression of SIRT1 and its deacetylase activity are repressed in normal cells by tumor suppressor proteins such as p53 [18], HIC1 [19], and DBC1 [20]. Inactivation of these tumor suppressor genes or activation of oncogenes such as E2F1 [21], c-Myc [22], and STAT5 [23] in pre-cancer and cancer cells enhance SIRT1 expression. SIRT1 is consistently upregulated in several primary solid tumors and hematopoietic malignancies, and promotes cell proliferation, angiogenesis, tumorigenesis and drug resistance [12, 13, 24]. Intriguingly, some other studies have shown that SIRT1 may act as a tumor suppressor. SIRT1 transgenic mice reduces the incidence of spontaneous carcinomas and the susceptibility to carcinogen-induced liver cancer [25] while SIRT1(+/−)/p53(+/−) mice have increased incidences of tumors in multiple tissues compared to SIRT1(+/+)/p53(+/−) mice. Treatment with resveratrol, which activates SIRT1, reduces tumorigenesis [26]. Together, these studies indicate complex, and possibly tissue-dependent roles of SIRT1 in both tumor promotion and suppression.

In this study, we have investigated the role of SIRT1 in KSHV-induced cellular transformation and tumorigenesis using the KMM model. We have found that KSHV infection upregulates the expression of SIRT1. We have further shown that SIRT1 is essential for KSHV-induced cellular transformation, and an inhibitor of SIRT1, nicotinamide (NAM), inhibits tumor progression and extends the survival of mice in a xenograft model of KSHV-induced tumorigenesis. These results identify SIRT1 as a putative oncogene and a potential therapeutic target of KSHV-associated tumors.

## RESULTS

### SIRT1 is upregulated in KSHV-infected cells

**Figure 1:**
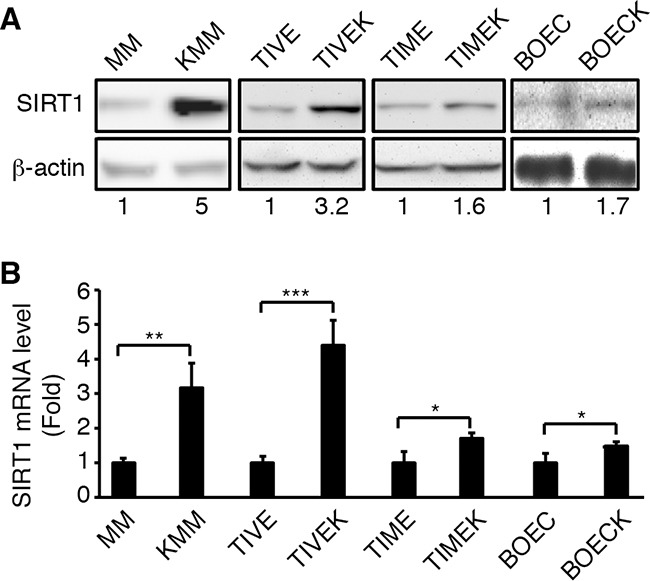
Upregulation of SIRT1 expression in different types of cells latently infected by KSHV **A**. Western-blotting analysis of SIRT1 protein expression. **B.** RT-qPCR analysis of SIRT1 mRNA expression. β-actin was used as an internal control. The numbers at the bottom of the panel are SIRT1 fold changes (A). The levels of uninfected cells are set as “1” for both protein and mRNA. Statistical analysis *p<0.05; **p<0.01; ***p<0.001.

SIRT1 is upregulated in several types of human cancer [12, 13, 24]. We examined the expression of SIRT1 in a number of cell types latently infected by KSHV. SIRT1 was upregulated at both protein and mRNA levels in MM cells, telomerase-immortalized human umbilical vein endothelial cells (TIVE), telomerase-immortalized human dermal microvascular endothelial cells (TIME), primary human blood outgrowth endothelial cells (BOEC) following KSHV infection (Figure 1A and 1B). KSHV-infected MM (KMM) and TIVE cells (TIVEK) are transformed while KSHV-infected TIME (TIMEK) and BOEC cells (BOECK) are not [5, 27–30]. Interestingly, the level of SIRT1 upregulation is higher in transformed cells than in non-transformed cells (3-fold *vs* < 2-fold). In addition, MM cells are primary cells and KSHV infection can cause immediate cellular transformation upon establishment of latency and expression of viral genes without going though any genetic alterations [5]. In contrast, TIVE cells were immortalized by telomerase. KSHV infection of TIVE cells did not lead to instant cellular transformation [28]. While TIVEK cells are transformed, they were selected from a single cell clone following long-term culture, which could contain genetic changes. In the remaining experiments, we used MM and KMM cells to examine SIRT1's role in KSHV-induced cellular transformation.

### Knockdown or knockout of SIRT1 suppresses cell proliferation and colony formation in soft agar of KSHV-transformed cells

**Figure 2:**
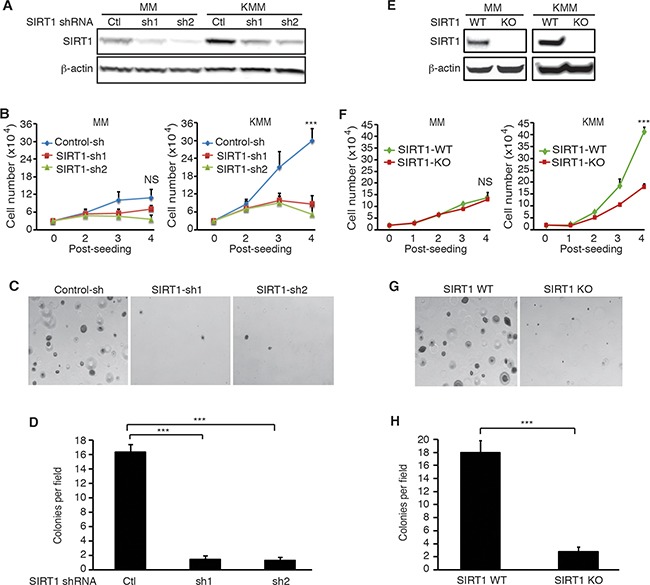
SIRT1 knockdown or knockout suppresses cell proliferation and colony formation in soft agar of KSHV-transformed cells **A.** MM and KMM cells were infected with lentiviruses harboring 2 different SIRT1 shRNAs (sh1 and sh2) or a scrambled control (Ctl), lysed at day 3 post-infection, and examined for knockdown efficiency by Western-blotting using β-actin as a loading control. **B.** MM and KMM cells with SIRT1 knockdown were counted at different time points after seeding. **C.** SIRT1 knockdown KMM cells at 2×10^4^ were suspended in 1 ml of 0.3% top agar and plated onto one well of 0.5% base agar in 6 well-plate at day 2 post-infection and maintained for about 3 weeks. Representative pictures of colonies were shown. **D.** The numbers of colonies from 9 random fields described in (C) were counted and the average colony number per field was shown as mean±s.d. **E.** Western-blotting analysis of SIRT1 in the wild-type (WT) and SIRT1 knockout (KO) cultures of MM and KMM cells using β-actin as a loading control. **F.** Wild-type and SIRT1 knockout MM and KMM cells were counted at different days post-seeding. **G.** Soft agar analysis of wild-type and SIRT1 knockout KMM cells. Representative pictures of colonies were shown. **H.** Colonies from 9 random fields described in (G) were counted and the average colony number per field was shown as mean±s.d. Statistical analysis ***p<0.001, NS, not significant.

To examine the role of SIRT1 in KSHV-induced cellular transformation, we performed knockdown of SIRT1 in MM and KMM cells with shRNAs targeting two different SIRT1 coding sequences (Figure 2A). As previously reported, KMM cells proliferated at a significantly faster rate than MM cells did [5]. SIRT1 knockdown dramatically inhibited cell proliferation of KMM cells while the effect on MM cells were marginal (Figure 2B). Both KMM and MM cells had similar proliferation rates following SIRT1 knockdown. Moreover, SIRT1 knockdown significantly inhibited the efficiency of colony formation of KMM cells in soft agar (Figure 2C and 2D). MM cells failed to form any colonies in soft agar with or without SIRT1 knockdown as they were not transformed [5]. To further confirm these results, we performed SIRT1 knockout using the CRIPSR/Cas9 system in both MM and KMM cells (Figure 2E). As expected, SIRT1 knockout dramatically inhibited cell proliferation in KMM cells while there was minimal effect on MM cells (Figure 2F). Moreover, SIRT1 knockout significantly decreased the efficiency of colony formation of KMM cells in soft agar (Figure 2G and 2H). Together, these results indicated that SIRT1 was required for optimal cell proliferation and colony formation in soft agar of KSHV-transformed cells.

Our previous study showed that SIRT1 knockdown caused a fraction of PEL cells (<10%) to undergo lytic replication [10]. We examined the viral lytic replication program in KMM cells following SIRT1 knockout. We did not detect expression of viral lytic proteins in any KMM cells following SIRT1 knockout (Supplementary Figure S1). These results are consistent with the fact that KMM cells are under tight viral latency [5]. Thus, the effects of SIRT1 knockdown or knockout on cell proliferation and colony formation in soft agar of KSHV-transformed cells were unlikely due to the reactivation of viral lytic replication.

### Knockdown and knockout of SIRT1 induces cell cycle arrest in KSHV-transformed cells

**Figure 3:**
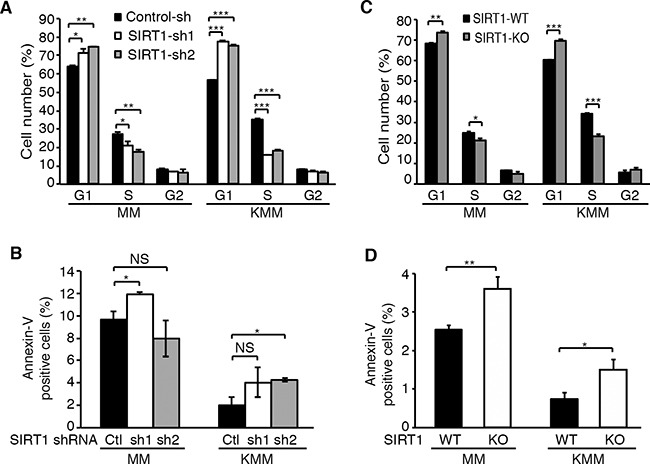
SIRT1 knockdown or knockout induces cell cycle arrest but has minimal effect on apoptosis in KSHV-transformed cells **A.** MM and KMM cells with SIRT1 knockdown as described in Figure 2 were pulsed with 10 μM BrdU for 2 h, stained with a pacific blue-conjugated anti-BrdU antibody and propidium iodide, and analyzed by FACS for cell cycle progression. **B.** MM and KMM cells with SIRT1 knockdown were stained with an anti-Annexin V antibody and analyzed by FACS for Annexin V-positive cells. **C.** Cell cycle analysis of wild-type and SIRT1 knockout cultures of MM and KMM cells as described in (A). **D.** Analysis of Annexin V-positive cells in wild-type and SIRT1 knockout cultures of MM and KMM cells as described in (B). Statistical analysis *p<0.05; **p<0.01; ***p<0.001, NS, not significant.

Because knockdown and knockout of SIRT1 suppressed cell proliferation and colony formation in soft agar of KSHV-transformed cells, we examined the effects on cell cycle and apoptosis. Cells were transduced with SIRT1 shRNA lentiviruses, split at day 2 post-transduction, pulsed with 10 μM BrdU for 2 h at day 4 post-transduction, and examined for cell cycle progression. SIRT1 knockdown induced cell cycle arrest of KMM cells at G1 phase by increasing G1 phase cells from 58% to 75-78%, and decreasing S phase cells from 35% to 16-18% (Figure 3A). The effect of SIRT1 knockdown on cell cycle progression of MM cells were more subtle, increasing G1 phase cells from 65% to 71-75%, and decreasing S phase cells from 28% to 19-21% (Figure 3A). In contrast, the effects of SIRT1 knockdown on apoptosis were marginal in both MM and KMM cells. SIRT1 knockdown only increased the number of apoptotic cells from 2% to 4-5% in KMM cells (Figure 3B). One shRNA increased the number of apoptotic cells from 10% to 12% while the second one decreased it from 10% to 8% in MM cells (Figure 3B). Similar results were obtained following SIRT1 knockout. SIRT1 knockout increased G1 phase cells from 60% to 70% and decreased S phase cells from 34% to 23% in KMM cells, while it increased G1 phase cells from 68% to 74% and decreased S phase cells from 25% to 21% in MM cells (Figure 3C). The effect of SIRT1 knockout on apoptosis of both MM and KMM cells were also subtle by increasing the number of apoptotic cells from 2.5% to 3.6% in MM cells and from 0.8% to 1.5% in KMM cells (Figure 3D). Thus, SIRT1 knockdown or knockout mainly affected the cell cycle progression of MM and KMM cells rather than apoptosis, and the effect on KMM cells was stronger than on MM cells.

### SIRT1 knockout induces contact inhibition and cyclin-dependent kinase inhibitor 1B (p27^Kip1^) expression

**Figure 4:**
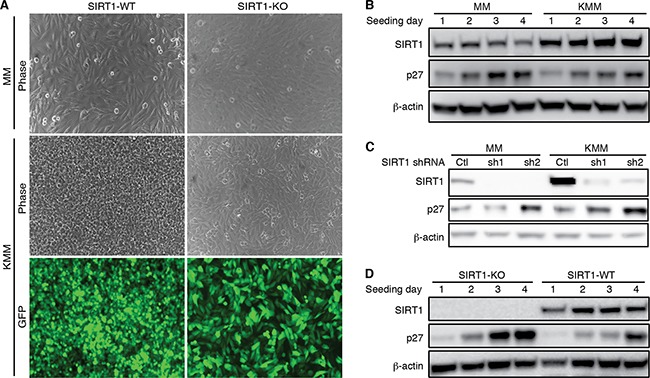
SIRT1 knockout induces contact inhibition and p27 expression **A.** Wild-type (WT) and SIRT1 knockout (KO) MM and KMM cells were seeded at the same cell numbers at about 50% confluency and cultured for 10 days with daily change of media. At day 10, representative pictures were taken and shown. Contact inhibition was observed in MM cells with or without SIRT1 knockout. KMM cells lost contact inhibition as shown by the overlapping cells in culture. SIRT1 knockout induced contact inhibition in KMM cells. **B.** MM and KMM cells were seeded at 30% confluency and lysed at day 1, 2, 3 and 4 post-seeding. The cultures reached 50%, 80%, 100% confluency at day 1, 2, 3 post-seeding. The expression levels of SIRT1 and p27 proteins were examined using β-actin as a loading control. **C.** MM and KMM cells were infected with lentiviruses of 2 different SIRT1 shRNAs (sh1 and sh2) or a scrambled control (Ctl), lysed at day 3 post-infection, and examined for the expression of SIRT1 and p27 proteins by Western-blotting using β-actin as a loading control. **D.** Wild-type and SIRT1 knockout KMM cells were seeded at 30% confluency and lysed at day 1, 2, 3 and 4 post-seeding. The cultures reached 50%, 80%, 100% confluency at day 1, 2, 3 post-seeding. The expression levels of SIRT1 and p27 proteins were examined using β-actin as a loading control.

The inhibitory effect of SIRT1 knockdown or knockout on cell proliferation only became obvious after day 2 post-seeding in KMM cells (Figure 2B and 2F). Furthermore, SIRT1 knockdown or knockout reduced the efficiency of colony formation in soft agar (Figure 2C and 2G). Therefore, we further examined the role of SIRT1 in contact inhibition. As previously reported, MM cells were contact inhibited while KMM cells were not (Figure 4A) [5]. SIRT1 knockout rendered KMM cells contact inhibited (Figure 4A), indicating that SIRT1 mediated contact inhibition in KMM cells.

Contact inhibition increases the expression of cyclin-dependent kinase inhibitor 1B (p27^Kip1^), which binds to cyclin-CDK (cyclin-dependent kinase) complexes to induce cell proliferation arrest in G1 cell cycle phase [31]. Indeed, the p27 protein level was increased in MM cells when the cells became confluent (Figure 4B). In this experiment, the culture was seeded at 30% confluency, which reached 50%, 80% and 100% confluency at day 1, 2, and 3 post seeding, respectively. Induction of p27 was also observed in wild-type KMM cells but at a lower level (Figure 4B). Importantly, SIRT1 knockdown increased p27 level in KMM cells to level similar to that in MM cells (Figure 4C). Similarly, SIRT1 knockout increased p27 level in KMM cells (Figure 4D). Thus, while contact inhibition induced p27 protein expression in MM cells, upregulation of SIRT1 in KMM cells inhibited the induction of p27. As a result, SIRT1 knockdown in KMM cells induced cell proliferation arrest and contact inhibition, and inhibited the efficiency of colony formation in soft agar.

### p27 knockdown rescues the inhibitory effects of SIRT1 knockdown and knockout on cell proliferation and colony formation in soft agar

**Figure 5:**
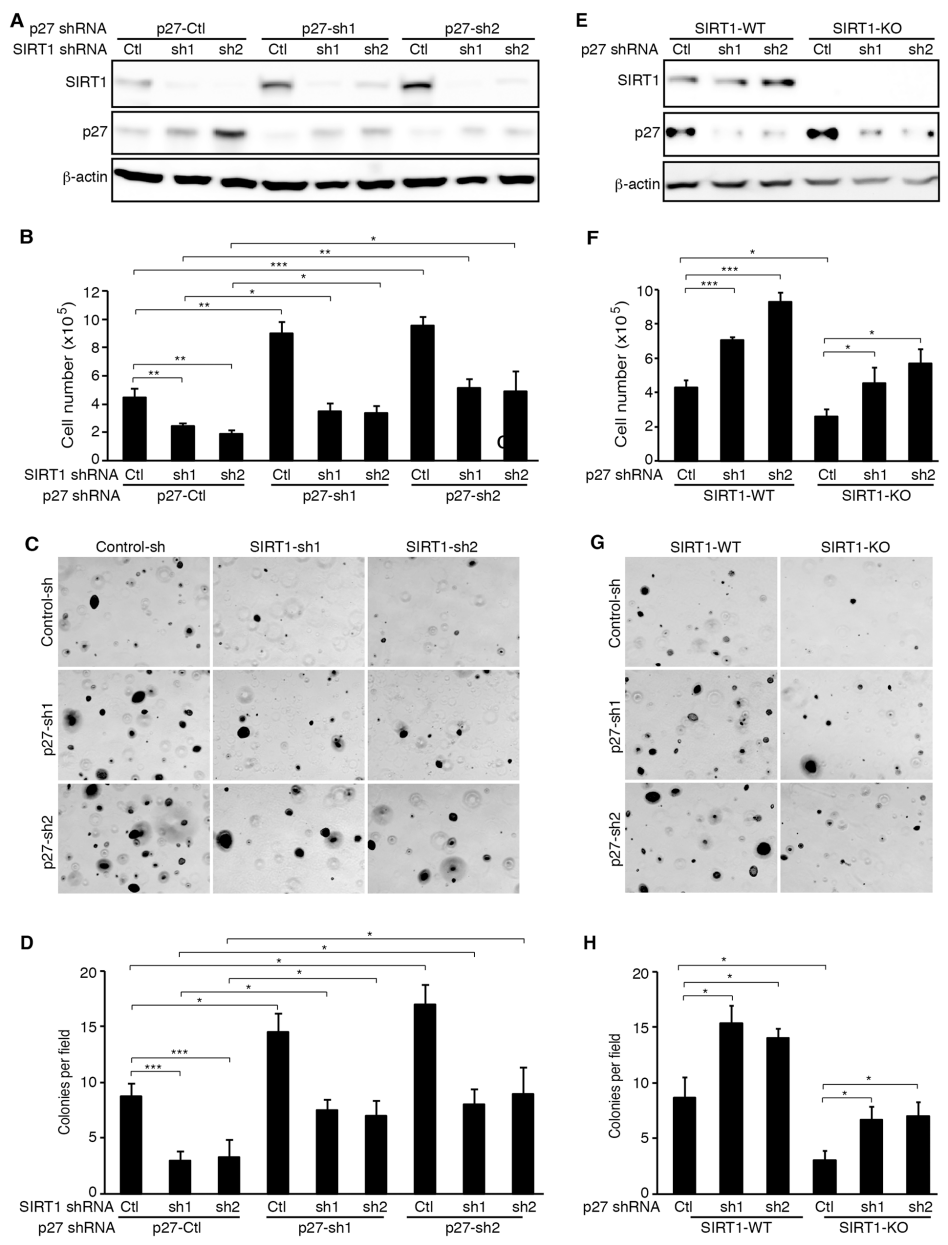
p27 knockdown rescues the inhibitory effects of SIRT1 inhibition on cell proliferation and colony formation of KSHV-transformed cells **A.** KMM cells were infected with lentiviruses harboring 2 different p27 shRNAs (sh1 and sh2) or a scrambled control (Ctl), and selected with blasticidin for 1 week. The stable p27 knockdown cells were further infected with lentiviruses harboring 2 different SIRT1 shRNAs (sh1 and sh2) or a scrambled control (Ctl), lysed at day 3 post-infection, and examined for SIRT1 and p27 knockdown efficiencies by Western-blotting using β-actin as a loading control. **B.** KMM cells with SIRT1 and p27 knockdown were counted at day 2 post-infection. **C.** KMM cells at 2×10^4^ with SIRT1 and p27 knockdown at day 2 post-infection with the SIRT shRNA lentiviruses as described in (A) were suspended in 1 ml of 0.3% top agar and plated onto one well of 0.5% base agar in 6 well-plate and maintained for about 3 weeks. Representative pictures of colonies were shown. **D.** The numbers of colonies from 9 random fields described in (C) were counted and the average colony number per field was shown as mean±s.d. **E.** Wild-type (WT) and SIRT1 knockout (KO) KMM cells were infected with lentiviruses harboring 2 different p27 shRNAs (sh1 and sh2) or a scrambled control (Ctl), lysed at day 3 post-infection and examined for the expression of SIRT1 and p27 proteins by Western-blotting using β-actin as a loading control. **F.** Wild-type and SIRT1 knockout KMM cells with and without p27 knockdown were counted at day 2 post-infection. **G.** Soft agar analysis of wild-type and SIRT1 knockout KMM cells with and without p27 knockdown. Representative pictures of colonies were shown. **H.** Colonies from 9 random fields described in (G) were counted and the average colony number per field was shown as mean±s.d. Statistical analysis *p<0.05; **p<0.01; ***p<0.001, NS, not significant.

To determine the role of p27 up-regulation in the inhibitory effects of SIRT1 knockdown and knockout on cell proliferation and colony formation, we performed p27 knockdown. We first established stable p27 knockdown KMM cells expressing 2 different p27 shRNAs or a scrambled control shRNA (Figure 5A). We then infected stable cells with lentiviruses harboring SIRT1-specific shRNAs or a scrambled control. At day 2 post-infection, we observed efficient knockdown of SIRT1 (Figure 5A). As expected, SIRT1 knockdown inhibited cell proliferation by 50-60% in cells stably expressing the p27 scrambled control shRNA (Figure 5B). However, cells with simultaneous knockdown of SIRT1 and p27 regained the proliferation rate. Interestingly, among SIRT1 scrambled control shRNA transduced cells, stable knockdown of p27 also had higher proliferation rates than their stable p27 control knockdown cells, suggesting suboptimal suppression of p27 by SIRT1 in KMM cells (Figure 5B). The results of colony formation in soft agar were consistent with those of cell proliferation (Figure 5C and 5D). SIRT1 knockdown inhibited the efficiency of colony formation in cells stably expressing the p27 scrambled control shRNA; however, cells with simultaneous knockdown of SIRT1 and p27 regained the efficiency of colony formation (Figure 5C and 5D). Again, among SIRT1 control shRNA scrambled transduced cells, stable knockdown of p27 had more robust colony formation efficiencies than their stable p27 control knockdown cells (Figure 5C and 5D).

To further confirm the role of p27 up-regulation in the inhibitory effects of SIRT1 knockdown on cell proliferation and colony formation, we transduced the SIRT1 knockout cells with p27 shRNAs or the scrambled control. We examined the expression levels of SIRT1 and the efficiencies of p27 knockdown at day 2 post-transduction (Figure 5E). As expected, knockdown of p27 significantly reduced the inhibitory effect of SIRT1 knockout on cell proliferation (Figure 5F) and the efficiency of colony formation in soft agar (Figure 5G and 5H). Again, p27 knockdown also increased cell proliferation and the efficiency of colony formation in KMM cells (Figure 5F-5H). Interestingly, the protein expression of SIRT1 was upregulated in the stable p27 knockdown cells (Figure 5A), which was confirmed in transient knockdown KMM cells with one p27 shRNA (Figure 5E). However, further experiments are required to confirm these results. Taken together, the results indicated that p27 upregulation mediated the inhibitory effects of SIRT1 knockdown or knockout on cell proliferation and colony formation in soft agar.

### Pharmaceutical inhibition of SIRT1 inhibits cell proliferation, colony formation in soft agar and tumor formation *in vivo* of KSHV-transformed cells

**Figure 6:**
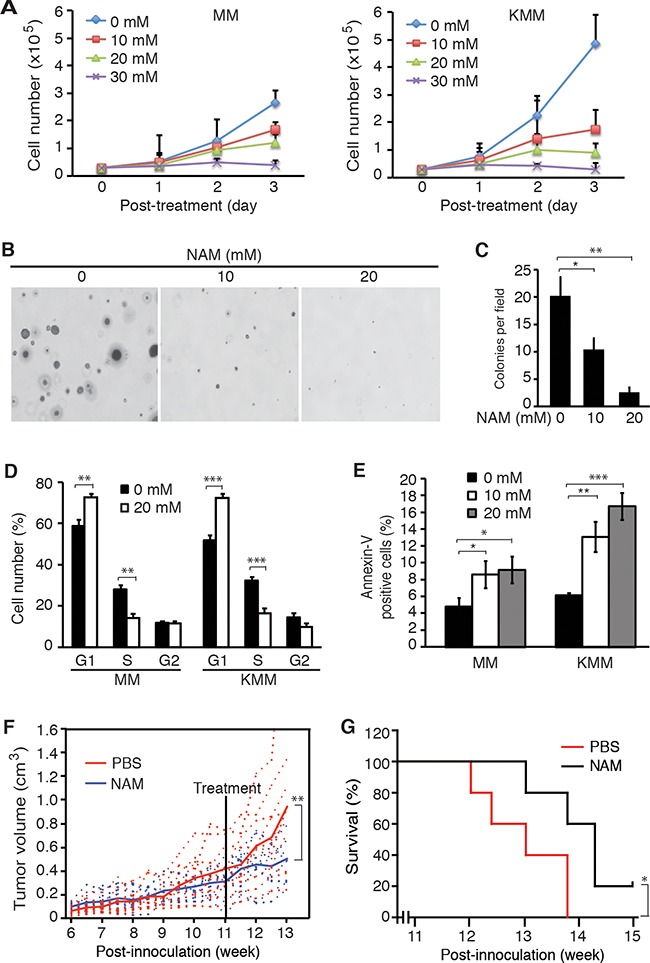
SIRT1 inhibitor NAM suppresses cell proliferation and colony formation *in vitro*, and extends the survival of mice *in vivo* **A.** MM and KMM cells were treated with different concentrations of NAM, and the numbers of cells were counted at different time points as indicated. **B.** KMM cells at 2×10^4^ were seeded in the soft agar with different concentrations of NAM and cultured in medium with the same concentrations of NAM in the soft agar for 3 weeks. Representative pictures of colonies were shown. **C.** The numbers of colonies from 9 random fields described in (B) were counted and the average colony number per field was shown as mean±s.d. **D.** MM and KMM cells treated with 20 mM NAM for 48 h were collected for cell cycle analysis. **E.** MM and KMM cells treated with 10 mM and 20 mM of NAM for 72 h were collected for apoptosis analysis. **F.** KMM cells at 10^7^ were subcutaneously injected into each flank of mice. Tumor volumes were monitored twice a week. When the average tumor sizes reached 0.4 cm^3^, half of the mice were treated daily with NAM at 300 mg/kg while the other half were treated with PBS by IP injection. Tumor volumes, calculated as length×width×height, were shown with the dash lines representing the volumes of individual tumors while the solid line representing the average tumor volumes. **G.** Kaplan-Meier survival analysis of mice treated with NAM or vehicle control PBS. Statistical analysis *p<0.05; **p<0.01; ***p<0.001.

Both knockdown and knockout of SIRT1 suppressed cell proliferation and colony formation in soft agar of KSHV-transformed cells, indicating SIRT1 could be a putative therapeutic target for KSHV-induced tumorigenesis. We examined the effect of NAM, a general inhibitor of sirtuins [32], on KSHV-transformed cells. Treatment with NAM inhibited cell proliferation of KMM cells in a dose-dependent and time-dependent manner (Figure 6A). NAM also inhibited the proliferation of MM cells but with less effect, particularly at lower doses. At 10 mM, NAM inhibited the proliferation of KMM cells by 65% and MM cells by 35% at day 3 post-treatment. NAM also dramatically inhibited the efficiency of colony formation of KMM cells in soft agar (Figure 6B and 6C). NAM induced cell cycle arrest in both MM and KMM cells. Treatment with NAM at 20 mM increased G1 phase cells from 59% to 73% and decreased S1 phase cells from 28% to 14% in MM cells while it increased G1 phase cells from 51% to 74% and decreased S1 phase cells from 33% to 17% in KMM cells (Figure 6D). NAM also induced low levels of apoptosis in both MM and KMM cells. NAM at 10 and 20 mM increased the number of apoptotic cells from 5% to 8.6% and 9.2%, respectively, in MM cells, and from 6.1% to 13.1% and 16.8%, respectively, in KMM cells (Figure 6E). The effect of NAM on apoptosis on both MM and KMM cells were stronger than those observed following SIRT1 knockdown or knockout, which might be due to its off-target effect.

Having observed the inhibitory effect of NAM on KSHV-transformed cells, we further examined NAM's effect on KSHV-induced tumorigenesis. We subcutaneously injected 10^7^ KMM cells into each flank of 4-week old athymic homozygous nude mice and monitored the tumor volume growth. Tumors were observed by week 5-6 post-inoculation (Figure 6F). We treated the mice with NAM by intraperitoneal injection at 300 mg/kg daily at week 11 post-inoculation when the average tumor size reached 0.4 cm^3^. NAM inhibited the growth of tumors (Figure 6F) and significantly increased the survival of the mice (Figure 6G, *P* = 0.0431).

## DISCUSSION

**Figure 7:**
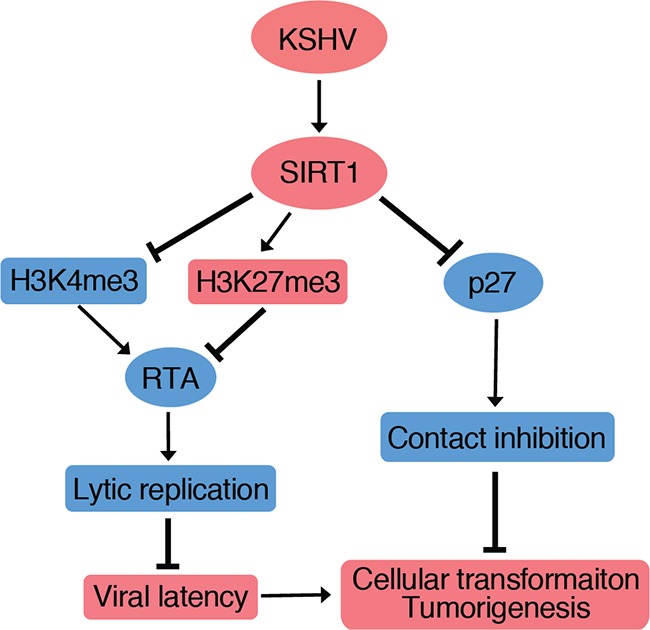
A model illustrating SIRT1 promotion of cellular transformation through two independent mechanisms (1) SIRT1 abolishes cell contact inhibition by suppressing p27 expression; and (2) SIRT1 inhibits RTA expression to promote viral latency by increasing H3K27me3 repressive mark and decreasing H3K4me3 active mark on the RTA promoter [10, 11].

In this study, we showed that SIRT1 was upregulated at both mRNA and protein levels in several cell types latently infected by KSHV. In KSHV-transformed cells, SIRT1 was required for cell proliferation and cellular transformation as knockdown or knockout of SIRT1 induced cell cycle arrest and inhibited colony formation in soft agar. We also showed that a general inhibitor of sirtuins, NAM, inhibited the proliferation and cellular transformation of KSHV-transformed cells. In vivo, NAM inhibited the progression of KSHV-induced tumors, and extended the survival of mice in a KS-like model. Together, these results have identified SIRT1 as a putative oncogene and a potential therapeutic target of KSHV-induced tumors. We have previously shown that SIRT1 inhibits KSHV lytic replication and the expression of viral lytic genes including RTA by epigenetic remodeling of viral chromatin, and direct inhibition of RTA transactivation of other viral lytic genes [10, 11]. Thus, SIRT1 contributes to KSHV-induced cellular transformation and tumorigenesis by performing dual functions, *i.e.* by promoting viral latency, and by enhancing cell proliferation and overriding contact inhibition (Figure 7).

Despite SIRT1's oncogenic property in KMM cells, overexpression of SIRT1 alone in MM cells was insufficient to affect cell proliferation and induce loss of contact inhibition (data not shown), indicating that dysregulation of other cellular genes and pathways by KSHV oncogenes is also required for inducing cellular transformation of MM cells.

Mechanistically, we showed that SIRT1 suppressed the expression of p27 to promote cell proliferation and overcome contact inhibition as SIRT1 knockdown or knockout upregulated the expression of p27 protein, and induced cell cycle arrest and contact inhibition. Furthermore, knockdown of p27 was sufficient to rescue the inhibitory effect of SIRT1 knockdown and knockout. As SIRT1 is a metabolic sensor, our results provide further evidence that KSHV reprograms the metabolic pathways to promote cell proliferation and cellular transformation, in this case, by upregulating SIRT1 to inhibit p27 [7].

p27 is a member of a family of CKIs and plays an important role in fundamental cellular processes and human malignancies [33]. p27 inhibits cell proliferation and induces contact inhibition; hence it is frequently inactivated in human cancers through accelerated proteolysis [34]. Downregulation of p27 is associated with poor prognosis in a variety of human malignancies, whereas restoration of p27 level inhibits tumor growth and progression [33, 34]. Because of the importance of dysregulating p27 in cancers, identification of new mechanisms that regulate its expression and function could lead to better understanding of the molecular basis of tumorigenesis and developing novel cancer therapies.

Although it has been shown that p27 expression is primarily regulated at the post-transcriptional level [35], the upstream regulators of p27 remain largely unknown. Thus, our results have identified SIRT1 as a novel upstream regulator of p27, hence it could be a putative therapeutic target. Indeed, a number of studies have shown that SIRT1 is a putative oncogene and targeting SIRT1 is effective in inhibiting tumor growth and progression of liver cancer and chronic myeloid leukemia among others [13, 36, 37]. Our results have also shown that pharmaceutical inhibition of SIRT1 is an effective approach in inhibiting KSHV-induced tumorigenesis.

In a previous study, we have shown that KSHV vCyclin inactivates p27 by inducing its cytoplasmic relocalization at contact-inhibited high-density condition to promote KSHV-induced cellular transformation [8]. Results from the current study showed that SIRT1 regulated p27 at the post-transcriptional level but not transcriptional level. However, we did not observe any changes of p27 cytoplasmic relocalization following SIRT1 knockdown or knockout (data not shown), indicating that SIRT1 might regulate p27 level through the nuclear degradation pathway. SIRT1 regulates numerous cellular processes by deacetylating transcriptional factors and their co-activators such as p53 [36], RelA/p65 [38], FOXO [17], and p300 [39]. Thus, SIRT1 could directly or indirectly regulate p27 through these transcriptional factors and co-activators. Furthermore, other factors, such as SKP2 and CKS1, regulate the ubiquitination and proteasome degradation of p27 [40–42]; hence SIRT1 could also directly or indirectly regulate p27 stability through these factors.

A recent study has shown that p27-mediated contact inhibition deactivates the mammalian target of rapamycin (mTOR) pathway, resulting in the suppression of the senescence program [43]. KSHV infection induces mesenchymal transition and loss of contact inhibition [5, 44, 45], which might contribute to the constitutive activation of the mTOR pathway in KSHV-infected cells [46], and hence the susceptibility of KS tumors to rapamycin [47]. vCyclin induces loss of contact inhibition at high cell density [8], which might contribute to its ability to induce senescence [48] and activation of the mTOR pathway [46]. Similarly, SIRT1 mediates KSHV inhibition of p27 and contact inhibition could also contribute to the activation of the mTOR pathway and induction of senescence. Interestingly, vCyclin induction of senescence is blocked by vFLIP, which is required for the proliferation of KSHV-infected cells [48]. Whether vFLIP is required to block SIRT1-mediated senescence in KSHV-transformed cells remains to be further investigated.

Our results have shown that SIRT1 is upregulated in a number of cell types latently infected by KSHV. During viral latent infection, only a handful of KSHV latent genes are expressed. These include LANA, vFLIP, vCyclin and a cluster of miRNAs. Further studies are required to identify the viral genes and cellular pathways that mediate KSHV-induced upregulation of SIRT1.

In this study, NAM dramatically inhibited cell proliferation and colony formation in soft agar. In a KSHV-induced KS-like tumor model, NAM inhibited tumor progression and significantly increased the survival of mice. NAM, the amide of vitamin B3, also known as nicotinic acid or niacin, is the product of sirtuins co-substrate NAD, which is a feedback inhibitor of sirtuins' activity [49]. NAM has very low toxicity *in vitro* and *in vivo* compared to other small molecule inhibitors. However, NAM inhibits all seven members of sirtuins (SIRT1-7) [49]. NAM is also a co-substrate of poly (ADP-ribose) polymerases (PARPs), hence manifests some other effects in addition to inhibition of sirtuins [50]. Furthermore, NAM is rapidly metabolized *in vivo*, presenting a challenge for maintaining an effective dose for inhibiting SIRT1's function *in vivo* [49]. It would be interesting to examine the effects of other more potent SIRT1 inhibitors in the KSHV-induced tumor model and explore their therapeutic potentials for KSHV-induced malignancies.

## MATERIALS AND METHODS

### Cell culture and reagents

MM and KMM cells, BOEC and BOECK cells, TIVE and TIVEK (also known as TIVE-LTC) cells, and TIME and TIMEK cells were obtained or cultured as previously described [5, 27–30]. 293T cells were maintained in Dulbecco's modified Eagle's medium (DMEM) with 10% fetal bovine serum (FBS, Thermo Fisher Scientific, Waltham, MA) and antibiotics (100 μg/mL penicillin and 100 μg/mL streptomycin). NAM was purchased from Sigma (Cat.# N0636, St. Louis, MO).

### Lentiviral shRNA knockdown

The SIRT1 shRNA constructs were prepared as previously described [27]. Briefly, sequences of SIRT1 shRNAs were inserted into the pLKO.1 lentiviral vector. The target sequences included shRNA1: GCACCGATCCTCGAACAATTC; shRNA2: GCAGGTTGCAGGAATCCAAAG; and scrambled shRNA: TTGTACTACACAAAAGTACTG. The p27-shRNAs constructs were purchased from GenTarget (San Diego, CA). The constructs contain a blasticidin selection marker and a RFP reporter cassette, which were used for selecting infected cells and monitoring infection rate, respectively. The sequences of the p27 shRNAs included shRNA1: CAAACTCTGAGGACCGGCAT; shRNA2: GACCAAATGCCTGACTCGTC; and scrambled shRNA: GTCTCCACGCGCAGTACATTT. Supernatants from 293T cells transfected with the shRNA and packaging constructs were collected at 60 h post-transfection followed by concentration of the virus at 24,000 rpm for 2 h at 4°C. The virus preparations were used for spinning infection of cells at 1,800 rpm for 45 min in the presence of 10 μg/ml of polybrene. Cells transduced with the lentivirus particles were examined for knockdown efficiency at day 3 post-transduction.

### CRISPR-Cas9 knockout

MM and KMM cells were first transduced with a lentiviral vector pLX-Cas9 expressing HA-tagged Cas9 and a blastcidin-resistant gene cassette, and selected with blastcidin at 10 μg/ml for MM cells and 20 μg/ml for KMM cells for 12 days. Stable cell cultures with Cas9 expression were then transduced with a lentiviral vector pLKO-SgRNA expressing a SgRNA and a puromycin-resistant gene at multiplicity of infection (MOI) 0.3, and selected with puromycin at 1 μg/ml for MM cells and 5 μg/ml for KMM cells for 4 days. Two SgRNAs were designed to target the N-terminal of rat SIRT1 SgRNA-1: 5'-CATGATTGGCACCGATCCTC-3' and SgRNA-2: 5'-CGGATAGGTATGCTTTAGGC-3'. A non-targeting SgRNA 5'-TGACATCAATTATTATACAT-3' was used as a control. We designed each SgRNA by using Cas-OFFinder program (http://www.rgenome.net/cas-offinder/) [51]. The resulting cells were counted and diluted to 5 cells per ml and seeded onto a 96-well plate with 100 μl per well so that approximately 50 cells were seeded into 1 plate. Individual cell seeding in most of the wells was confirmed by microscopy observation. Only clones from wells expanded from one cell were chosen to screen for SIRT1 expression by Western-blotting using an antibody against the C-terminal of rat SIRT1 protein (Cat.# 8469, Cell Signaling Technology, Danvers, MA). To further confirm the disruption of the gene in the selected clones, genomic DNA was extracted and used to amplify a fragment of 661 bp for SgRNA-1 and a fragment of 863 bp for SgRNA-2, respectively, spanning the targeting sites by PCRs. The PCR products were then sequenced using internal primers. In the cases of multiple sequence peaks, the PCR products were cloned into the pCR2.1 vector and approximately 10-20 bacterial colonies from each cell clone were selected for sequencing. Six SIRT1 knockout and 6 non-targeting (wild-type SIRT1) KMM clones were selected and pooled together, respectively. Similarly, 2 SIRT1 knockout and 2 non-targeting (wild-type SIRT1) MM clones were selected and pooled together, respectively, for further study.

### Soft agar assay

Soft agar assay was performed as previously described with minor modifications [5]. Briefly, a total of 2×10^4^ cells suspended in 1 ml of 0.3% top agar (Cat.# A5431, Sigma, St. Louis, MO) were plated onto one well of 0.5% base agar in 6 well-plates and cultured for 2-3 weeks. Colonies with a diameter > 50 μm were counted and photographed with a microscope.

### Cell cycle, BrdU incorporation and apoptosis assays

Cell cycle analysis were performed by staining with propidium iodide (Cat.# P4864, Sigma, St. Louis, MO). BrdU incorporation was performed by pulsing cells with 10 μM BrdU (Cat.# B5002, Sigma, St. Louis, MO) for 2 h followed by staining with a Pacific Blue conjugated anti-BrdU antibody (Cat.# B35129, Thermo Fisher Scientific, Waltham, MA). A PE-Cyanine 7 conjugated anti-Annexin V antibody (Cat.# 25-8103-74, eBioscience, San Diego, CA) was used to detect apoptotic cells following the instructions of the manufacturer. Flow cytometry was performed in a FACSCanto System (BD Biosciences, San Jose, CA). Analysis was performed with FlowJo (Treestar, Ashland, OR).

### Reverse transcription quantitative real-time polymerase-chain reaction (RT-qPCR)

Total RNA was isolated with the TRI Reagent (Cat.# T9424, Sigma, St. Louis, MO) according to the instructions of the manufacturer. Reverse transcription was performed with total RNA using Maxima H Minus First Strand cDNA Synthesis Kit (Cat.# K1652, Thermo Fisher Scientific, Waltham, MA). qPCR analysis was performed on Eppendorf Real Plexusing KAPA SYBR FAST qPCR Kits (Cat.# KK4602, Kapa Biosystems, Wilmington, MA). The relative expression levels of target genes were normalized to that of the internal control gene β-actin, which yielded 2^^-ΔΔCt^ values. The primers rat SIRT1-F: GAGTGTGCTGGAGGATCTGG and rat SIRT1-R: AGTGCTCTGATTTGTCTGGTGT were used for rat SIRT1; rat β-actin-F: CCATGTACCCAGGCATTGCT and rat β-actin-R: AGCCACCAATCCACACAGAG were used for rat β-actin; human SIRT1-F: GGACATGCCAGAGTCCAAGT and human SIRT1-R: CCCAAATCCAGCTCCTCCAG were used for human SIRT1; and human β-actin-F: ATCATTGCTCCTCCTGAGCG and human β-actin-R: CGGACTCGTCATACTCCTGC were used for human β-actin.

### Western-blotting analysis

Total cell lysates were separated in SDS polyacrylamide gels, electrophoretically transferred to nitrocellulose membranes (GE Healthcare, Pasadena, CA). The membranes were incubated sequentially with primary and secondary antibodies. The signal was developed using Luminiata Crescendo Western HRP substrate (Cat.# WBLUR0500, EMD Millipore, San Diego, CA). The antibodies used for Western-blotting included rabbit antibodies (mAbs) to SIRT1 (Cat.# 8469) and p27 (Cat.# 3686) from Cell Signaling Technology (Danvers, MA), and β-actin (Cat.# sc8432) from Santa Cruz (Santa Cruz, CA).

### Mouse model

The use of animals was approved by the University of Southern California Institutional Animal Care and Use Committee. Male athymic homozygous nude mice (4-weeks-old) were purchased from Harlan (Indianapolis, IN). KMM cells (10^7^ cells) were injected subcutaneously into each flank of both flanks of a mouse. Tumor volumes were monitored twice weekly and calculated according to the following formula (length×width×height). We started the NAM treatment when the average tumor size reached 0.4 cm^3^. Treatment was conducted by intraperitoneal injection of NAM at a dose of 300 mg/kg/daily. PBS was used as a vehicle control. The mice were terminated by CO2 inhalation when one of the tumors reached 1.2 cm^3^.

### Statistical analysis

For survival study, Kaplan-Meier survival analysis was performed and statistical significance was calculated using the log-rank test. For other data analysis, the two-tailed t-test or F-test of equality of variances were performed and *P* < 0.05 was considered statistically significant. Statistical symbols “*”, “**” and “***” represent *P* < 0.05, < 0.01 and <0.001, respectively, while “NS” indicates “not significant”.

## SUPPLEMENTARY FIGURE


