# Supplementary material for: SIRT1-mediated downregulation of p27Kip1 is essential for overcoming contact inhibition of Kaposi's sarcoma-associated herpesvirus transformed cells
Source: Oncotarget. 2016 Sep 30;7(46):75698–711. doi: 10.18632/oncotarget.12359 (PMC5342771; doi:10.18632/oncotarget.12359)
Supplement: Supplementary file 1 [file oncotarget-07-75698-s001.pdf]

SIRT1-mediated downregulation of $p27^{Kip1}$ is essential for overcoming contact inhibition of Kaposi's sarcoma-associated herpesvirus transformed cells

SUPPLEMENTARY FIGURE

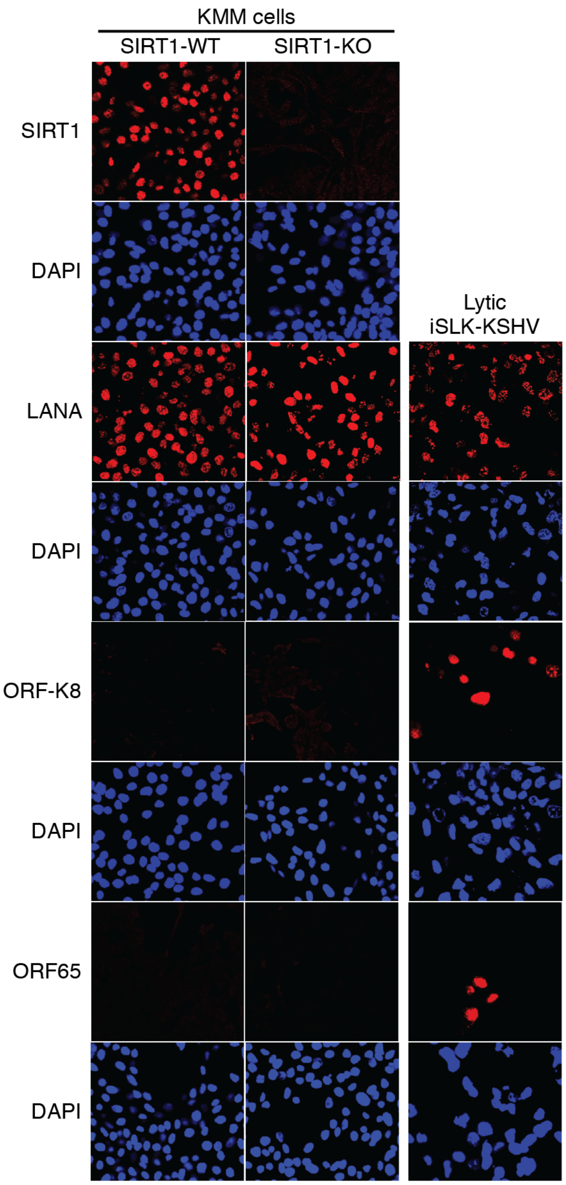

Supplementary Figure S1: Expression of KSHV proteins in wild-type KMM cells (SIRT1-WT) and KMM cells with SIRT1 knockout (SIRT1-KO). SIRT1-WT and SIRT1-KO KMM cells were stained for the expression of KSHV latent protein LANA, and lytic proteins ORF-K8 and ORF65 as well as SIRT1 protein. Cells were counter-stained with DAPI. KSHV-infected iSLK cells (iSLK-KSHV) were induced for lytic replication and used as positive controls.
